# A Multicenter Randomized Controlled Trial of Malignant Gastric Outlet Obstruction: Tailored Partially Covered Stents (Placed Fluoroscopically) versus Standard Uncovered Stents (Placed Endoscopically)

**DOI:** 10.1155/2014/309797

**Published:** 2014-12-24

**Authors:** Ding Shi, Feng Ji, Yin-su Bao, Yong-pan Liu

**Affiliations:** ^1^Department of Gastroenterology, The First People's Hospital of Yuhang District, No. 369 Yingbing Road, Hangzhou 311100, China; ^2^Department of Gastroenterology, The First Affiliated Hospital of Zhejiang University, Hangzhou 310003, China; ^3^Department of Gastroenterology, The First Affiliated Hospital of Henan College of Traditional Chinese Medicine, Zhengzhou 450000, China

## Abstract

The aim of our study is to compare the efficacy and safety of “outlet-shape” tailored stents with standard stents for the management of distal gastric cancer causing gastric outlet obstructions (GOOs) with varying gastric cavity shapes and sizes. To determine the shape and size of the GOOs, stomach opacifications were performed using contrast media before stenting. Two basic shapes of the residual cavity of the proximal GOO were observed: cup shaped or approximately cup shaped and funnel shaped or approximately funnel shaped. Other shapes were not found. In the GOO tailored group, the size and shape of the proximal ends of the tailored stent were suited for the residual cavity of the proximal GOO. The tailored stents included large cup-shaped stents and large funnel-shaped stents. GOO tailored covered stents led to less restenosis and reintervention rates compared to standard uncovered stents but with the same survival.

## 1. Introduction

Various types of metal stents are used for the treatment of nonresectable gastric outlet obstruction (GOO) [1–4]. Standard stents are similar to those to relieve esophageal obstructions. However, the gastric cavity caused by distal gastric cancer is frequently wide and therefore the ends of common stents with diameters ranging from 18 to 28 mm may not be appropriate for the natural shapes and sizes of the proximal GOO portions [5–9]. The too small proximal ends lead to migration of standard stents and might also be the cause of restenosis, because too small proximal ends have almost no effect on preventing ingrowth and overgrowth. Therefore, standard covered and uncovered stents have high migration and restenosis rates [10–14]. Although double- and triple-layer stents seem to prevent migration and tumor ingrowth [8, 15, 16], the shapes and sizes of the gastric cavities are usually not considered in these improved stents [15, 17]. Our hypothesis was that unresectable GOO tailored covered stents are superior to the standard uncovered ones in terms of stent reobstruction and stent migration. In the current study the efficacy and safety of GOO tailored covered stents for the treatment of nonresectable GOOs caused by distal gastric cancer were compared with standard uncovered stents.

## 2. Patients and Methods

This study was conducted between May 2009 and March 2013 and was designed as a multicenter, controlled, prospective, observational, and randomized clinical trial involving three large hospitals, the First People's Hospital of Yuhang District, the First Affiliated Hospital of Zhejiang University, and the First Affiliated Hospital of Henan College of Traditional Chinese Medicine. The study design was approved by the Infection Control and Ethics Committee of the above mentioned centers and was performed in compliance with the hospital policies related to the use of human subjects and human-derived material and informed consents regarding the study and procedures were obtained from all patients.

Inclusion criteria were (1) GOO defined by symptoms resulting in decreased oral intake (nausea, vomiting, and inability to eat), (2) the obstruction which was caused by primary distal stomach cancer, and (3) the site of stenosis which was between the gastric body and duodenum bulb. (4) All patients who were selected for stent placement had inoperable cancers or were unsuitable for surgery because of the presence of severe comorbid conditions. Exclusion criteria included the presence of only mild symptoms in patients who could tolerate a liquid diet, clinical evidence of perforation or peritonitis, and evidence of multiple small-bowel obstructions because of peritoneal seeding as well as diabetes or other diseases that affect gastric motility and use of promotility agents. Of the 75 patients, 10 patients refused stent implantation and the other 65 patients were randomly divided into two groups: a GOO tailored group (33 cases) in whom GOO tailored covered stents were used and a control group (32 cases) in whom uncovered standard stents were used. Patients were randomized to either the GOO tailored group or the control group, using a table of random numbers. Gastric outlet obstruction scoring (GOOS) was performed according to the scoring system introduced by Song et al. [8].

### 2.1. Stent Design

**Figure 1:**
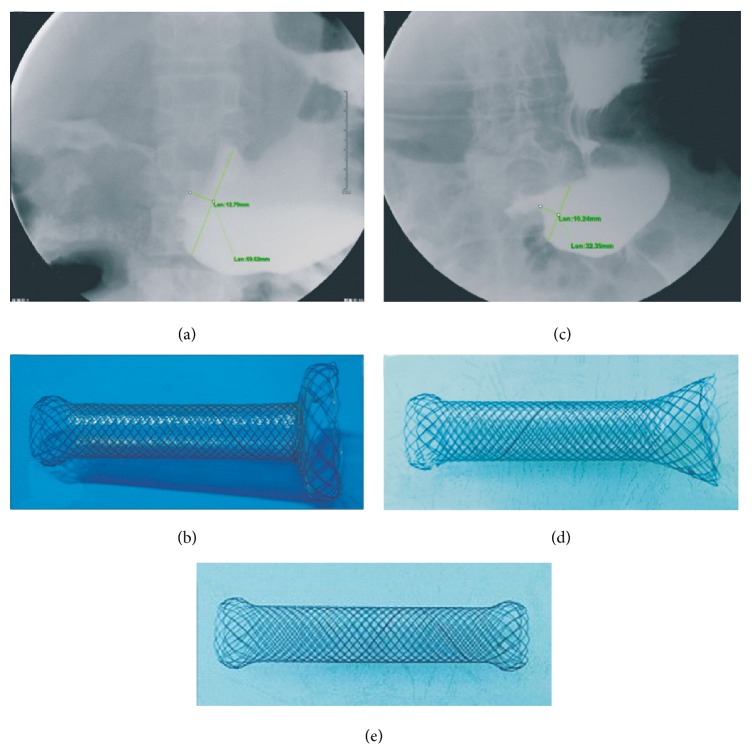
Examples of proximal GOO lumen shapes and images of GOO tailored stents for GOO. The distal portion of the GOO tailored stents was semispherical, with a length of 20 mm and a diameter of 28 mm. The middle segment had a diameter of 20 mm. The overall length of the stents was 100 mm. (a) A cup-shaped obstruction and (b) a cup stent. The proximal ends were large cup-shaped (53.3 mm ± 5.5 mm in diameter, 15 and 20 mm in length). (c) A funnel-shaped obstruction and (d) a funnel stent. The proximal ends were funnel-shaped (33.6 mm ± 3.6 mm in diameters, 25 mm and 30 mm in lengths). (e) Standard uncovered stent. The length of the stents was 100 mm and the ends were semispherical with diameters of 28 mm and length of 20 mm.

Stomach opacification was performed using contrast media less than three days before stent design in order to determine the shape of the GOO. Stomach opacification and stent design have been described by us previously [18]. Cup-shaped or approximate cup-shaped GOOs (Figure 1(a)) were found in 29 patients in each of the GOO tailored group and control group. Funnel-shaped or approximate funnel-shaped GOOs (Figure 1(c)) were found in four patients in the GOO tailored group and in three cases in the control group. The maximum breadth and length of the obstruction cup and funnel are shown in Table 1. Stents (Micro-tech (Nanjing) Co., Ltd, Nanjing, Jiangsu, China) (custom made) were designed to be cup-shaped (Figure 1(b)) or funnel-shaped (Figure 1(d)), according to the shapes of the proximal GOOs. The proximal ends of GOO tailored stents were large cup-shaped (53.3 ± 5.5 mm in diameter, 15 and 20 mm in length) and large funnel-shaped (33.6 ± 3.6 mm in diameters, 25 and 30 mm in lengths). The distal portion of the GOO tailored stents was semispherical, with a length of 20 mm and a diameter of 28 mm. The middle segment had a diameter of 20 mm. The overall length of the stents was 100 mm. Both the middle part and the bottom of the proximal cup segment and a part of the proximal funnel segment were covered by a polyethylene membrane. The rest of the stents were not covered. The stents were mounted on a delivery system with an outer diameter of 6 mm and an overall length of 130 to 180 cm.

Standard uncovered stents MTN-CG-s-20/100 (Micro-tech (Nanjing) Co., Ltd, Nanjing, Jiangsu, China) were used in the control group. The ends of the stents were semispherical with diameters of 28 mm and length of 20 mm. The length of the stents was 100 mm (Figure 1(e)).

### 2.2. Procedure

**Figure 2:**
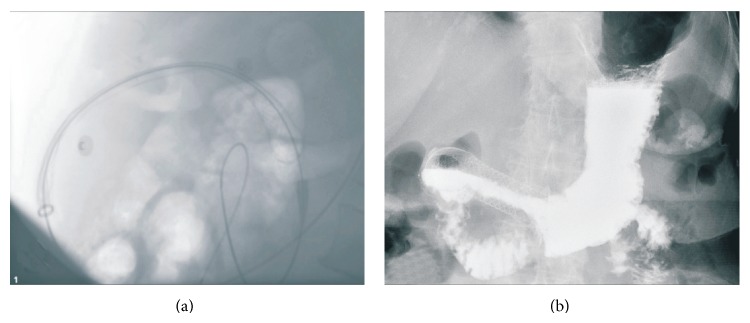
X-ray images of stents. (a) Image showing a stent released. (b) An example of barium contrast radiography of a funnel stent.

All stent placements were performed by the same gastroenterologists. The stent cups with a proximal diameter of 53.3 mm ± 5.5 mm at full expansion were available in 2 lengths, 15 and 20 mm, which corresponded to obstructions <15 mm and ≥15 mm in length. The stent funnels with a proximal diameter of 33.6 ± 3.6 mm were also available in 2 lengths, 25 and 30 mm, which corresponded to obstruction lengths of <25 mm or ≥25 mm. The overall length of GOO tailored stents was 100 mm. GOO tailored stents were implanted by a peroral method under fluoroscopic guidance without the use of endoscopy. The patients took a left lateral decubitus position without sedation, anesthesia, or airway intubation. Endoscopy was first performed to locate the lesion and place the guidewire (MTN-Qf-90/42-b, Micro-tech (Nanjing) Co., Ltd, Nanjing, Jiangsu, China) which was used in the process of tailored stent implantation because of its strong support force, followed by withdrawal of the endoscope and insertion of the delivery system (MTN-CR-6.0/180, Micro-tech (Nanjing) Co., Ltd, Nanjing, Jiangsu, China) per os over the guide wire. It was important to maintain the position of the guidewire while the delivery system was inserted. If the proximal end of the tailored stent did not completely fit into the residual antral wall, the recycling thread of the stent was pulled up by endoscopy, or an ERCP balloon catheter was used to push the stent forward in order to adjust the location of the stent. The whole procedure was performed under fluoroscopic guidance (Figure 2(a)) [19, 20]. The standard uncovered stents were implanted by a through-the-scope method [6, 21]. In brief, the delivery system (MTN-CR-3.3/160, Micro-tech (Nanjing) Co., Ltd, Nanjing, Jiangsu, China) was passed over a guide wire (Jagwire, Boston Scientific, Natick, MA, USA) through the working channel of the endoscope after the guide wire was confirmed to be located in the intestinal lumen. If a stent was found to be insufficient to traverse the whole stricture segment, a second regular stent was implanted, overlapping the first stent.

### 2.3. Followup

Three to five days after stent placement, barium contrast radiography was performed to document the position and the function of the stents (Figure 2(b)). Fluorouracil-based chemotherapy was given to patients according to the wishes of the patients' families if patients' physical conditions were satisfactory. Monthly telephone calls were made to assess food intake until patients' deaths. For scheduled follow-up visits every three months, patients came to the hospital for examination until death. A follow-up barium study or endoscopy was carried out only in the patients with recurrent symptoms.

### 2.4. Outcome Measurements

Primary endpoints were the stent complications ingrowth/overgrowth and stent migration. Secondary endpoints were the adverse events including bleeding, abdominal pain and food impaction.

### 2.5. Statistical Analyses

According to previous literature, reobstruction and migration occurred in 49.2% of self-expanding metal stents particularly in GO patients [22]. A minimum sample size calculation with this data and a statistical power of 0.95 revealed a necessary sample size of at least 20 patients per group (15% losses during followup included). Statistical analyses were performed using SPSS for Windows (version 11.0. Chicago, SPSS Inc.). Continuous variables were compared with Student's *t*-test while categorical variables were compared with the chi square test and Fisher' test. A *P* value <0.05 was considered to be statistically significant.

## 3. Results

### 3.1. Patient Characteristics

Table 2 shows that both groups were similar in terms of demographic variables, degree of differentiation, TNM staging, chemotherapy, and comorbidities. Because most of malignant GOOs occurred as preterminal adverse events in advanced gastric carcinoma, chemotherapy was performed in only 3 of 65 patients. There were no statistical differences using the balanced test. In the GOO tailored group, there were twenty-nine patients with cup-shaped stents and four patients with funnel-shaped stents. Thirty-two patients in the control group received standard uncovered stents. The whole stricture segment could be traversed by one stent in all patients and no second stents were used.

### 3.2. Technical and Clinical Outcome

The efficacy and complication rates of the two groups are shown in Table 3. Technical success is defined by accurate stent placement in the targeted lesion site. In the GOO tailored group one-cup stent could not be implanted successfully because of stent delivery system looping into the dilated gastric fundus, and the guide wire could not be passed across the stricture in one patient in the standard uncovered stent group. All stents were transpyloric. In one patient of the GOO tailored group, a proximal funnel stent initially protruded into the wide gastric cavity because of inaccurate implantation but was adjusted to fit into the residual antral cavity.

Clinical success was determined by resolution of obstructive symptoms and the ability to restart a low residue diet after stent placement. Two patients did not show improvement of obstruction symptoms in each group. There was no statistical difference in terms of symptom improvement. Follow-up contrast studies showed that the stents were completely open in the GOO tailored group, and there was poor expansion of stents in two patients of the control group.

### 3.3. Stent Complications

**Figure 3:**
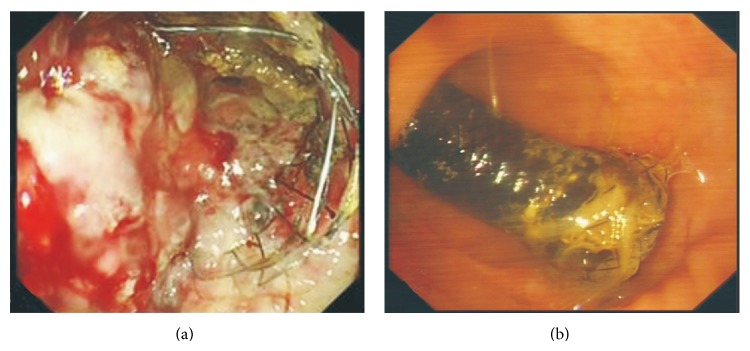
Images of stents taken by endoscopy. (a) An example of standard uncovered stent obstruction caused by tumor ingrowth and overgrowth. (b) An example of a funnel-shaped GOO tailored stent that had partly migrated into the stomach.

Stent obstruction caused by tumor ingrowth or overgrowth (Figure 3(a)) appeared in seven patients in the control group and one patient in the GOO tailored group during the follow-up period. Tumor ingrowth appeared in the distal uncovered section of one GOO tailored stent. In cases of restenosis, a standard uncovered stent was reinserted to overlap a primary stent, and the symptoms resolved in all eight patients. The time to develop proximal partial stent migration (Figure 3(b)) in two patients of the GOO tailored group was found to be 258 and 313 days. No stent migration was found in the control group. There was no statistical difference in terms of stent migration between the two groups. In the cases of stent migration, replacement stents were reimplanted after removal of the former ones.

### 3.4. Adverse Events

Bleeding and abdominal pain occurred in the two groups, but the incidence of bleeding and abdominal pain in the tailored group was significantly higher than that in the control group, respectively (Table 3); however, they were mild and did not need special treatment. Food impaction after stent placement was treated endoscopically.

During the follow-up period after stent placement, 62 patients died. The mean survival time was 231 ± 23 days (range 30–387 days) in the GOO tailored group and 212 ± 22 days (range 43–267 days) in the control group. One patient in each group failed to return for followup.

## 4. Discussion

Stenting has been the preferred treatment method for inoperable malignant GOO caused by gastric cancer [23–25]. However, stent migration and restenosis remain the main deficiencies of standard stents [4, 6, 8, 17, 26–30]. Although improved stents have been used to treat malignant GOOs, the migration rates have remained high (9.1% and 10%) [7, 15]. There were no migrations in the control group but the migration rate of GOO tailored stents in the current study was 6.3%, which is lower than previous reports. Moreover, the current study did not find a statistical difference in terms of stent migration between the GOO tailored group and the control group. The large stent cup or funnel may have contributed to the nonoccurring migration. However, cases of partial stent migration into the stomach were found which may have been related to the stent covering and the length. The stent cup (or funnel) prevents stent migration distally but not proximally into the stomach. Because the lengths of stents were not individually designed, the body of some stents may have been longer than the stenotic area. Unlike other covered stents [8, 26], the tailored stents proximally migrated into the stomach. Although proximal migration is a deficiency of GOO tailored stents, these proximally migrated stents can be easily removed and replaced. From this perspective, the proximal stent migration is easier to handle than distal migration.

Restenosis even in recently improved stents has been reported to range from 8% to 10.3% [7, 31]. The restenosis rate of GOO tailored stents in the current study was 3.2%, which is lower than in the above reports. However, ingrowth as a result of tumor progression occurred in the distal uncovered stent section in one patient in the GOO tailored group and in seven patients of the control group. The current result demonstrated that GOO tailored stents (partly covered) were superior to standard uncovered ones in preventing ingrowth. This result is consistent with previous reports [6, 28, 30]. Uncovered stents are accepted as standard for the treatment of malignant GOOs [11, 16] because they are effective in preventing stent migration and tend to remain patent longer than covered stents. In addition, covered stents are associated with a more frequent need for reintervention than uncovered stents [20, 32]. Although covered stents can decrease restenosis by tumor ingrowth, this advantage is offset by their higher migration rate which has been reported to range from 6.9% to 27.3% [6, 8, 20, 26, 30]. However, unlike standard uncovered stents, we did not find restenosis of proximal ends of the GOO tailored stents, presumably because the big stent cups and funnels provided a wider space to accommodate tumor ingrowth and the covered part of the cup and funnel provided a barrier to tumor overgrowth. In the current study, two patients did not show improvements of symptoms after stent placement, which might be due to functional gastric outlet obstruction due to neural involvement of the tumor [15, 33, 34].

A limitation of this study was the lack of individualization of stent lengths. Larger studies using stents in which the lengths are also GOO adjusted are planned for the future.

In summary, using covered GOO tailored stents for gastric cancer treatments led to significant less restenosis compared with uncovered standard stents, whereas the migration rate was not significantly different. The reintervention rate was also significantly less in the covered GOO tailored stent group. Mild adverse events like bleeding and abdominal pain were less in the conventional stent group but did not need interventions. Length variation needs further improvement for GOO tailored stents.

## Figures and Tables

**Table 1:** Numbers of cases and obstruction dimensions.

	GOO tailored group		Control group		*P* value
	Cup obstruction (*n* = 29)	Funnel obstruction (*n* = 4)	Cup obstruction (*n* = 29)	Funnel obstruction (*n* = 3)	
Breadth (mm)	53.3 ± 5.5	33.6 ± 3.6	53.5 ± 5.7	33.9 ± 3.7	>0.05
Length (mm)	15.1 ± 1.5	17.4 ± 1.8	15.3 ± 1.6	17.4 ± 1.9	>0.05

**Table 2:** Patient characteristics.

	GOO tailored (*n* = 33)	Control (*n* = 32)	*P* value
Male/female	18/15	17/15	>0.05
Average age (y)	76.4 ± 7.7	75.8 ± 7.6	>0.05
Differentiated degree			>0.05
Moderately	10	9	
Poorly	23	23	
TNM staging			>0.05
IIIA	6	8	
IIIB	8	6	
IV	19	18	
Comorbidities	4	3	>0.05
Chemotherapy	2	1	>0.05
GOOSS (mean)	4.4 ± 0.4	4.2 ± 0.4	>0.05

Lost for followup	1	1	

TNM, tumor, nodes, metastasis; GOOSS, gastric outlet obstruction score.

**Table 3:** Efficacy and complications.

	GOO tailored group	Control group	*P* value
Technical success	96.9%	96.9%	>0.05
Clinical success	93.8%	93.5%	>0.05
GOOSS change	3.2 ± 0.5	3.1 ± 0.4	>0.05
Ingrowth + overgrowth	1	7	<0.05
Migration	2	0	>0.05
Adverse events			
Bleeding	11	2	<0.05
Abdominal pain	13	1	<0.05
Food impaction	1	1	>0.05
Reintervention rate	9.4%	22.6%	<0.05
Survival (d)	231 ± 23	212 ± 22	>0.05

GOOSS, gastric outlet obstruction score.
